# Synthetic pectin–cellulose nanofiber capsule recapitulates the mechanical properties of a regenerating plant cell wall

**DOI:** 10.1073/pnas.2528515123

**Published:** 2026-06-01

**Authors:** Cyril Grandjean, Ravi Shanker, Sarah A. Pfaff, Anran Mao, Jordi Chan, Sophie Asnacios, Atef Asnacios, Sulin Zhang, Daniel J. Cosgrove, Enrico Coen, Anna J. Svagan, Pauline Durand-Smet

**Affiliations:** ^a^ Université Paris Cité, CNRS, Matière et systèmes complexes, Paris F-75013, France; ^b^ Department of Fibre and Polymer Technology, Kungliga Tekniska högskolan Royal Institute of Technology, Stockholm 100 44, Sweden; ^c^ Department of Biology, Pennsylvania State University, University Park, PA 16802; ^d^ Department of Cell and Developmental Biology, John Innes Centre, Norwich Research Park, Colney Lane, Norwich NR4 7UH, United Kingdom

**Keywords:** primary cell wall, protoplasts, cellulose, mechanical properties, synthetic walls

## Abstract

Plant primary cell walls are dynamic supramolecular assemblies composed of layered cellulose, hemicellulose, and pectin, progressively built through synthesis and secretion. However, the specific architectural features and structural components sufficient to endow the mechanical properties of the wall remain unclear. Here, we construct a minimal synthetic spherical shell and compare its structural and mechanical properties to those of a plant single-cell system. To eliminate complexities from intercellular connectivity and developmental history, we exploit the ability of plant protoplasts to regenerate cell walls de novo. Compression tests of regenerating protoplasts between parallel plates reveal that wall stiffness increases with wall thickening over time. Despite differences in assembly pathways, architecture, and composition, the synthetic shell exhibits a similar thickness-dependent modulus and similar material stiffness. The synthetic shell, mainly composed of pectin and cellulose nanofibers, mirrors the mechanical behavior of regenerating primary cell walls, suggesting that these components play a major role in conferring key mechanical properties in the limit of compressive small deformations. Extending this comparative approach should allow similarities and differences in component interactions in controlling wall behavior to be identified.

## Significance

Plant growth and shape are controlled by the mechanics of primary cell walls. Nevertheless, the minimal features required to provide strength remain unclear. Here, we compare the structure and mechanics of regenerating cell walls in plant protoplasts—single cells initially deprived from their walls—with those of a synthetic wall. We show that assemblies with increasing numbers of layers of cellulose nanofibers and pectin recapitulate the increase in stiffness observed in natural walls during thickening. Thus, basic wall mechanics emerge from simple material combinations. This comparative approach provides a tractable platform for exploring similarities and differences between synthetic and native walls guiding bioinspired design in the future.

Plant primary cell walls are composed of multiple layers arranged in a cross-lamellate architecture. Each layer consists of interwoven polysaccharide networks of cellulose, hemicellulose, and pectin that are progressively assembled through cellulose synthesis at the plasma membrane and matrix secretion from Golgi-derived vesicles (1–7). Cell wall composition and architecture are shaped not only by developmental history (8) but also by mechanical stresses imposed during growth (9–15). The result is a heterogeneous complex three-dimensional hydrated polymer network (16).

This structural complexity raises a fundamental question: which features of the wall contribute to its mechanical resilience vs. other aspects such as growth, signaling, metabolite transport, or protection from pathogens and environmental stressors (17, 18). One approach to address this question is through genetic or enzymatic perturbations, using mutants defective in wall biosynthesis or applying enzymes that degrade or modify specific wall polymers (19–24). These studies have provided valuable insight into the role of various wall components (pectin, cellulose, and xyloglucan), by quantifying how these perturbations modify aspects of wall mechanics such as elasticity, viscoelasticity, loosening, or softening under various loading conditions. However, while such studies can identify necessary components that contribute to wall mechanics, they do not establish sufficiency. A synthetic approach provides a complementary strategy. If complexity is essential for wall mechanical properties, a simple synthetic wall would not be expected to recapitulate the mechanics of a native wall. Alternatively, if complexity is required for functions other than mechanics, a synthetic wall could readily capture mechanical properties but not other features.

Early efforts to synthetically mimic wall mechanics involved cellulose-based bacterial films made of bacterial cellulose nanoribbons grown in hemicellulose or pectin solutions (25, 26). The resulting composites showed much lower stiffness (27, 28) than native plant cell walls (29). An alternative strategy involves layer-by-layer (Lbl) assembly of capsule walls made of alternating adsorbed nanolayers of wood-derived cellulose nanofibers (CNFs) and pectin, a method that offers precise control of composition and thickness (30). Although such capsules have previously been reported (31–33), their mechanical properties remain uncharacterized, and no direct comparison has been made with native cell walls.

A convenient method for assessing the mechanical properties of synthetic capsules is parallel-plate compression (34), a technique applied to both animal and plant cells (35–37). Here, we use this approach to quantitatively compare two systems: regenerating cell walls in plant protoplasts and CNF-pectin synthetic capsules. If native wall architectural features, such as cross-linking or cross-lamellar organization, are essential for mechanical properties, we expect the synthetic capsules would be unable to capture the mechanical properties of plant cell walls.

Wall regeneration has been studied in both *Arabidopsis thaliana* and *Nicotiana tabacum* (BY2) (38–40) cell lines. Cellulose architecture during wall regeneration has been visualized in protoplasts derived from *Arabidopsis* rosette leaves using advanced microscopy (38, 41, 42). Mechanical characterization of intact BY2 cells (43, 44) and *Arabidopsis* isolated cells showed that turgor pressure dominates cell stiffness (36, 45). However, the mechanical properties of the regenerating walls remain poorly quantified. We here focus on BY2 protoplasts because their capacity to regenerate and proliferate in suspension culture make them ideal for monitoring dynamic changes in cell wall mechanics.

In this study, we integrate structural (Atomic Force Microscopy, AFM and Electron Transmission Microscopy, TEM), composition, and mechanical (viscoelastic) analyses to directly compare synthetic primary cell walls with regenerating walls of BY2 protoplasts. At selected stages during regeneration, we assess surface structure via AFM scanning, wall compositions with polysaccharide staining and mass spectroscopy, and mechanical properties using compression assays. Our results show that synthetic layered CNF-pectin walls can recapitulate mechanical characteristics of regenerating primary cell walls, suggesting that complexity is not essential for capturing key mechanical properties of native cell walls.

## 1. Results

### 1.1. Regenerating a Cell Wall vs. Building a Synthetic Wall.

**Scheme 1.**
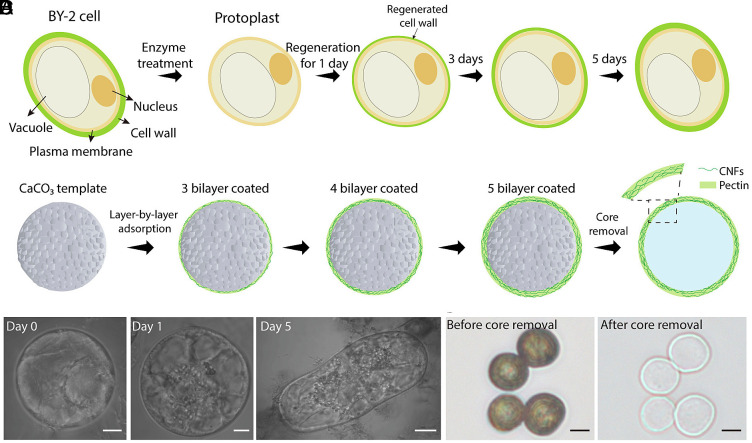
(*A*) Regeneration of cell wall in BY2 protoplasts and (*B*) bottom–up assembly of synthetic walls by adsorption of pectin and CNFs layers on top of CaCO_3_ template particles. (*C*) The shapes of BY2 protoplasts after 0,1 and 5 d of cell wall regeneration. (*D*) The template of synthetic capsules was removed using acid and washed with MilliQ-water, yielding a core-shell structure with water-filled core and multilayered wall. [Scale bar, 5 µm (*C* and *D*) or 10 µm (*C*, day 5).]

To investigate de novo cell wall formation, we first generated wall-less plant cells, protoplasts, from BY2 *N. tabacum* cells in liquid culture. Protoplasts were obtained by a combination of cell wall degradation and hypo-osmotic shock (details in *Materials and Methods*). Protoplasts were cultured in primary cell wall regeneration medium for 5 consecutive days (Scheme 1 *A* and *C*). On day 0, immediately after protoplasting, protoplasts were spherical, averaging 38.3 ± 3.2 μm in diameter, mean ± SD (*SI Appendix,* Fig. S3 and Scheme 1*C*). After 5 d in the regeneration medium, most cells lost their spherical shape (Scheme 1*C*), indicating that a new cell wall had formed. By this time, their average diameter increased to around 55 ± 13 μm (*SI Appendix,* Fig. S3).

Synthetic cell walls were prepared via the bottom–up Lbl assembly protocol, involving the alternate adsorption steps of pectin and cationically modified CNFs onto calcium carbonate (CaCO_3_) microparticle templates, as schematically illustrated in Scheme 1*B*. Each bilayer (bL) comprises one layer of pectin followed by one layer of CNFs and the total number of bilayers in the walls varied from 3 to 6. These multilayered structures constitute polyelectrolyte multilayers, also referred to as polyelectrolyte complexes, which form spontaneously when oppositely charged macromolecules interact (46). The pectin carried a negative charge due to its carboxyl groups, while the surfaces of the CNFs were rendered positively charged through the covalent introduction of quaternary ammonium moieties. The assembly of polyelectrolyte complexes is primarily driven by the entropic release of counterions and water molecules upon complex formation (47). The cationic charge on the CNFs was needed in the Lbl assembly. Following Lbl assembly, the CaCO_3_ template was dissolved using citric acid solution, and the residual salts were removed via thorough washing with MilliQ-water, finally yielding a core-shell structure with a water-filled interior (Scheme 1 *B* and *D*). The final size of the 6 bL capsules after core removal was 15.8 ± 2.7 μm in diameter (*SI Appendix,* Fig. S3), about half that of protoplasts. Table 1 provides a summary comparison of the regenerating and synthetic walls. As discussed below, although xyloglucan and mannan were not intentionally included in the synthesis of the bL capsules, the synthetic walls contained trace amounts of these components, likely originating from impurities in the pectin and CNF used for bL assembly.

**Table 1.** Comparison between structural and mechanical properties between the synthetic and regenerated cell walls

Property	Synthetic wall (Lbl capsules)	Regenerated wall (BY2 cells)
Diameter	≈15.8 ± 2.7 µm (6bL)	≈55 ± 13 µm (day 5)
Morphology	Spherical	Spherical (day 0) → elongated (day 5)
Wall assembly	Layer-by-layer adsorption	Natural synthesis, wall secretion
Wall composition	Cellulose (CNFs), Pectin, Xyloglucan, Mannan	Cellulose microfibrils, Pectin, Xyloglucan, Mannan
Covalent cross-links between wall polymers	No (electrostatic, hydrogen bonding, van der Waals interactions).	Yes, in addition to noncovalent cross-links, hydrogen bonding, van der Waals, electrostatic interactions.
Wall architecture	Multilayered wall, random CNF orientation in layers	cross-lamellate cellulose microfibrils organization in layers
Cellulose distribution in wall	Homogenous	Heterogenous
Turgor	No turgor	Yes or No (when cells are plasmolyzed)
Growth	No	Yes
Wall thickness	≈70 to 180 nm (3bL to 5 bL)	120 to 250 nm (3 to 5 d)

### 1.2. Nanoscale Structural Comparisons.

**Fig. 1.**
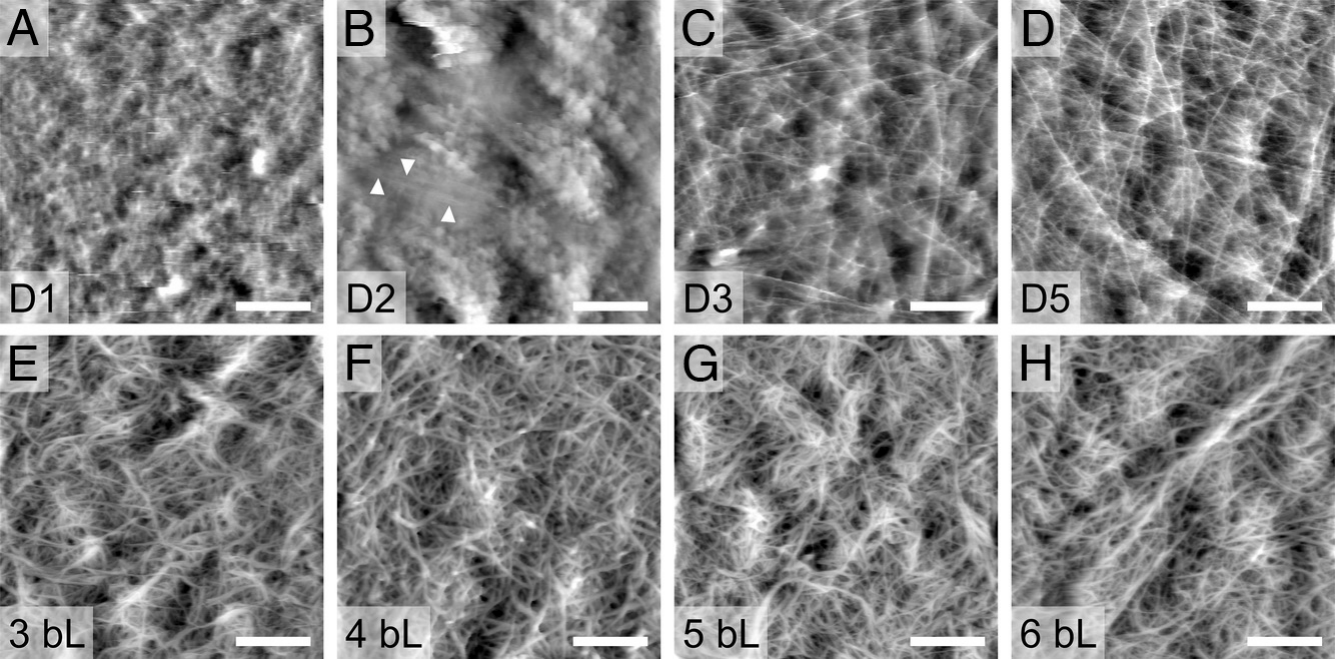
AFM of BY2 protoplasts and Lbl capsule surfaces. Regenerating protoplast (*A*) day 1, (*B*) day 2, (*C*) day 3, and (*D*) day 5 post harvest. Synthetic walls assembled with (*E*) 3 bL, (*F*) 4 bL, (*G*) 5 bL, and (*H*) 6 bL of pectin and CNFs. All panels show Height Sensor AFM channel (first-order plane fit, fifth order flatten). Arrows in *B* show fibers. (Scale bar, 500 nm.)

The nanoscale structure of the outermost cell wall layer in regenerating protoplasts at defined time points (post regeneration) was investigated with AFM. The same measurements were performed on hydrated synthetic walls of varying numbers of bilayers (Fig. 1). Progressive changes in surface ultrastructure were observed during wall regeneration in BY2 cells. At day 1, cellulose microfibrils were not discernible at the protoplast cell surface (Fig. 1*A*), whereas by day 2, nascent microfibrillar structures became evident (arrows in Fig. 1*B*), and became more numerous by day 3 and day 5 (Fig. 1 *C* and *D*). The absence of cellulose microfibrils at the surface of 1-d-old protoplasts was unexpected given that cellulose microfibrils are synthesized and extruded by cellulose synthase complexes embedded in the plasma membrane. Also, the outermost cell wall layer was expected to be similar at the different time points; 1, 2, 3, and 5 d. Cellulose can also be visualized using stains such as Calcofluor White. Adding Calcofluor to regenerating protoplasts revealed cellulose labeling from day 1 onward (Fig. 3 *H*–*K*), indicating there was not a delay in cellulose synthesis. The delayed detectability of cellulose microfibrils with AFM may reflect differences in the dynamics of cellulose deposition during wall regeneration. For instance, early stages are reported to involve the rapid diffusion of short fibrils, which then become consolidated into a relatively stable network of elongated microfibrils. As AFM scanning reveals surface features by interacting mechanically with the structure; the early unstable structure might be difficult to detect by this technique (42).

From day 3 onward, the characteristic cross-lamellate architecture of cellulose microfibrils in the primary cell wall became apparent and this distinct fibrillar morphology persisted through day 5, as shown in Fig. 1 *C* and *D*. From the micrographs, we observed that the integrated cellulose microfibrils organization was random.

Fig. 1 *E*–*H* presents AFM micrographs of the outermost surface layer of the synthetic walls that were composed of 3, 4, 5, or 6 bLs. The synthetic walls contained randomly oriented fibers, irrespective of the number of bilayers. The cross-lamellate structure was not replicated in the synthetic walls. Additionally, compared to the cellulose microfibrils observed in the regenerated walls (Fig. 1 *C* and *D*), the CNFs in the synthetic walls exhibited greater bundling. CNFs displayed a higher degree of curvature and lacked the extended, linear morphology of the cellulose microfibrils in the native cross-lamellate arrays (Fig. 1 *C* and *D*).

**Fig. 2.**
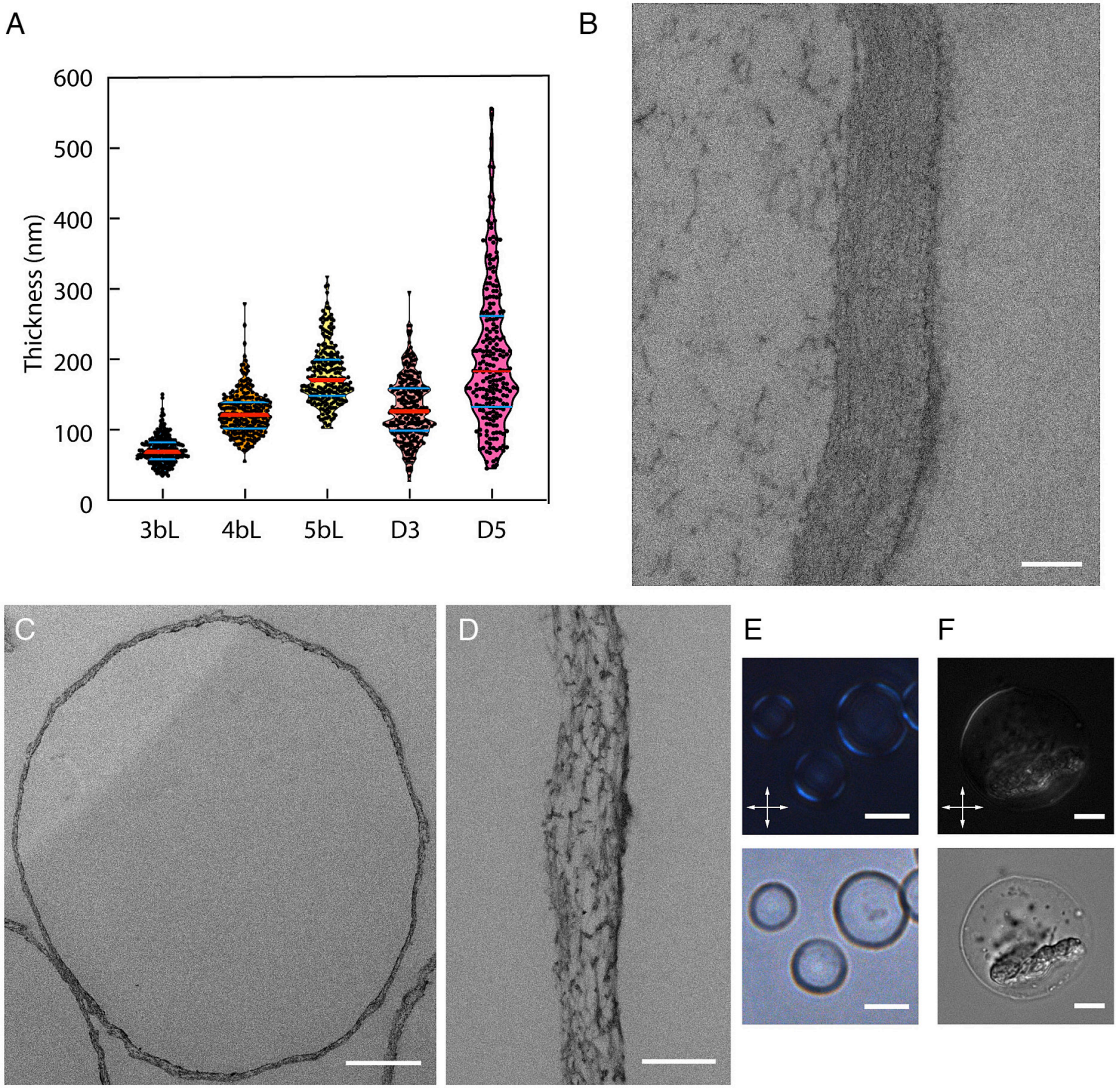
(*A*) Thicknesses measured from TEM of cross-sections of synthetic capsules (3bL, 4bL, and 5bL) and regenerated walls (day 3 and 5). (*B*) Cross-section of a regenerated wall (day 5). (*C*) Cross-section of a synthetic wall with 5 bilayers (3 and 4 bL TEM images are in *SI Appendix*). (*D*) close-up on the synthetic wall in (*C*). (*E* and *F*) POM (*Top*) and Brightfield (*Bottom*) images of a synthetic wall with 5 bilayers (*E*) and of plasmolyzed BY2 cell after 4 d of regeneration (*F*). [Scale bar, 200 nm (*B*), 2 µm (*C*), 200 nm (*D*), 10 µm (*E* and *F*).]

Wall thicknesses for regenerated and synthetic walls were evaluated from transmission electron microscopy (TEM) cross-sections (Fig. 2). Synthetic walls exhibit a uniform thickness throughout their structure (Fig. 2*C*). The appearance of Maltese cross patterns in the 5bL samples with polarized optical microscopy (POM) showed a birefringence signal suggesting a predominant layered ordering of the CNF (Fig. 2*E* and *SI Appendix,* Fig. S2). The regenerated cell walls also displayed a birefringence signal suggesting layered organization (Fig. 2*F*). The overall thickness of the synthetic walls increased with the number of bilayers: 71 ± 20 nm for 3bL, 124 ± 31 nm for 4bL, and 178 ± 42 nm for 5bL constructs (Fig. 2*A*). These thicknesses were matched by the thicknesses of regenerated BY2 cell walls (Fig. 2 *A* and *B*). The thickness of the regenerated walls increased with time: 130 ± 45 nm for day 3 and 204 ± 102 nm for day 5. While the absolute thickness values might be underestimated because TEM sample preparation inherently causes wall dehydration, wall thickening due to material accumulation was captured in both systems.

### 1.3. Wall Composition.

**Fig. 3.**
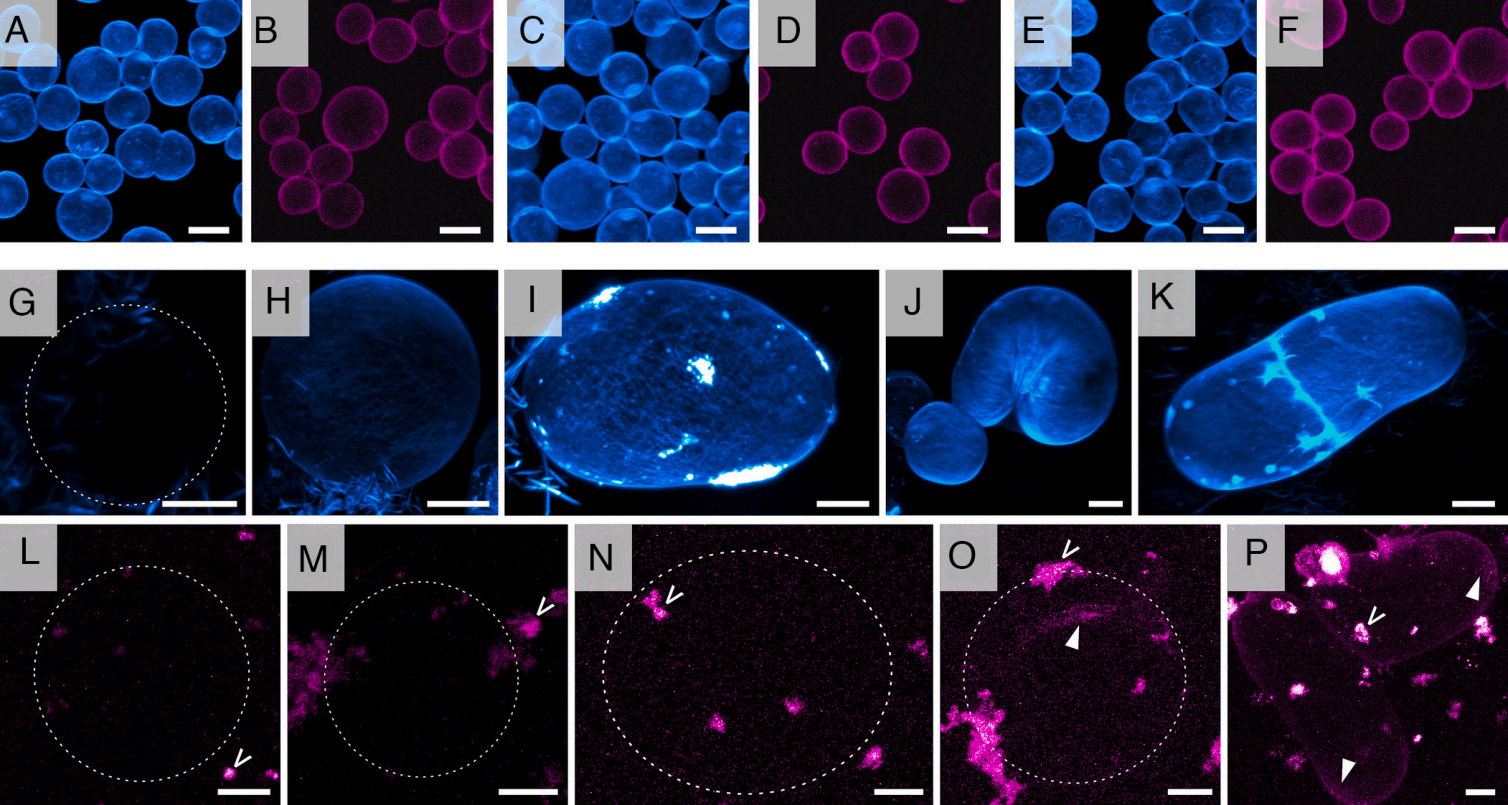
CLSM images of synthetic walls (*Upper* row) with 3bL (*A* and *B*), 4bL (*C* and *D*), or 5bL (*E* and *F*) and regenerating BY2 protoplasts (*G*-*P*) at day 0 (*G* and *L*), day 1 (*H* and *M*), day 2 (*I* and *N*), day 3 (*J* and *O*), and day 5 (*K* and *P*) of cell wall regeneration. The cellulose and pectin in synthetic walls and the regenerated cell walls were stained with Calcofluor white (CFW, cyan, in *A*, *C*, *E*, and *G*–*K*) and propidium iodide (PI, magenta, in *B*, *D*, *F*, and *L*–*P*). Dotted lines show cell outlines in images where the cell is not clearly visible. White arrowheads indicate PI signal coming from the cell wall and chevrons indicate aggregates of cell debris. (Scale bar, 10 µm.)

Cellulose and pectin in the regenerated and synthetic cell walls were stained with Calcofluor White (CFW) and propidium iodide (PI), respectively, and the resulting confocal laser scanning microscopy (CLSM) images are presented in Fig. 3 and *SI Appendix,* Fig. S4. As previously mentioned, BY2 protoplasts and the synthetic walls exhibited different size ranges. On day 0, immediately after protoplasting, the protoplasts showed neither CFW nor PI staining, confirming that the cell wall had been largely removed during the process. Staining confirmed the presence of the two main polysaccharides, cellulose and pectin, in the synthetic and regenerated walls (from day 1 to day 5). Cellulose staining was consistently homogeneous across all investigated synthetic wall bL numbers. By contrast, the regenerating walls of BY-2 cells showed a less uniform pattern, with brighter regions indicating areas of higher cellulose concentration. The intensity of cellulose staining increased from day 1 to day 5 of wall regeneration, suggesting that the cellulose network became more established and denser as regeneration progressed. This pattern may reflect a dynamic organization of the cellulose network, as previously described by Huh et al. (42). PI binds to -COOH groups and we observed that the fluorescence PI signal was detectable after day 3 for the native regenerating walls, indicating that either pectins were not synthesized and anchored in the early stages of regeneration (before day 3) or that there was change in the pectin’s molecular composition at day 3 (big patches of PI signal in Fig. 3 *L*–*P* come from cellular debris).

Gas chromatography–mass spectrometry (GC–MS) analysis was performed to investigate the monosaccharide composition of wall material, excluding crystalline cellulose. While imaging and staining approaches provide structural and spatial information, GC–MS enables sensitive detection of monosaccharides released from the hydrolyzable, noncrystalline fraction of the wall. Importantly, the reported monosaccharide compositions (*SI Appendix,* Table S1) reflect only the TFA-hydrolyzable fraction of the wall and therefore represent only a subset of the total wall material, as some pectic domains are resistant to TFA hydrolysis. For regenerated BY2 cell walls, accurate determination of wall dry weight was not possible due to the extremely small amount of material recovered. Consequently, monosaccharide composition was expressed as mole fractions (mol%), rather than normalized to wall dry weight, to avoid introducing substantial quantitative uncertainty. Synthetic capsule data are provided for comparison. Monosaccharide compositions of synthetic capsules and regenerated BY2 cell walls at day 5 are reported in *SI Appendix*, Table S1. Regenerated walls exhibited a heterogeneous monosaccharide composition dominated by glucose, mannose, galactose, and arabinose, each contributing approximately 20 to 25 mol% of the detected monosaccharide pool, whereas uronic acids (galacturonic acid), rhamnose, and xylose were present only as minor components. In contrast, synthetic capsules were dominated by pectic material and glucose-based polymers. Several additional monosaccharides were consistently detected at lower molar fractions, with galactose being the most abundant among these minor components. These additional monosaccharides likely originate from residual nonpurity of the starting materials: commercial pectins are heterogeneous and not chemically pure, and cellulose-derived materials originating from wood may retain trace amounts of secondary cell wall components. Although the relative abundances of monosaccharides type differed substantially between the synthetic and regenerated walls, the types of monosaccharides present were largely similar between the two systems.

### 1.4. The Wall of Regenerating Protoplasts Stiffens as the Wall Thickens.

**Fig. 4.**
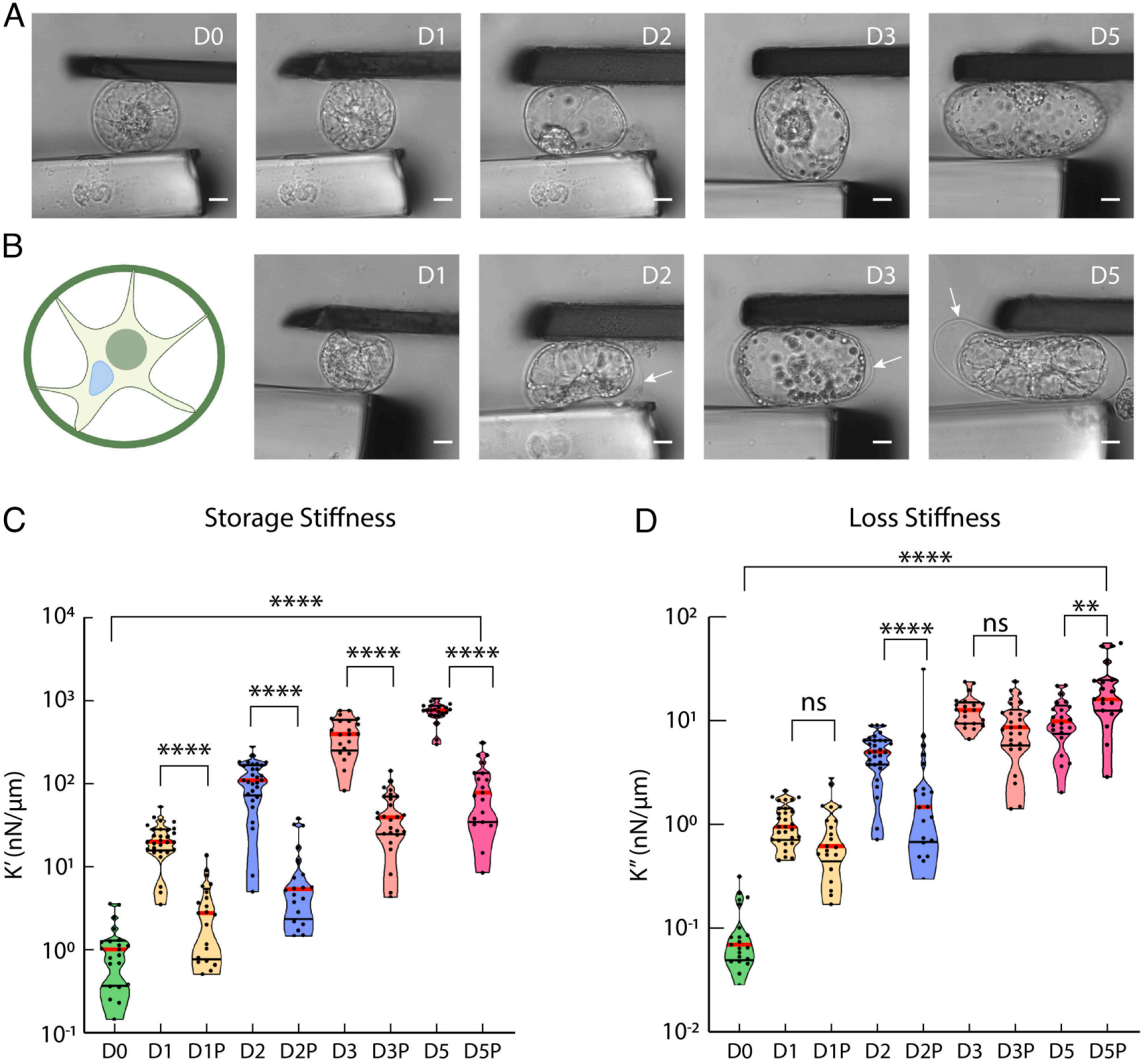
Storage and loss stiffnesses for regenerating BY2 cells. Light microscopy images of cells at each day of regeneration, in both control (*A*) and plasmolyzed (*B*) conditions, (Scale bar, 10 µm.) White arrows indicate the cell wall in plasmolyzed condition. (*C*) storage stiffness (K’) and (*D*) loss stiffness (K’’) are plotted as a function of the days of cell wall regeneration. **P-value < 0.01, ****P-value < 0.0001 Mann–Whitney test. D0 and D5P ****P-value < 0.0001 Kruskal–Wallis test (Dunn’s multiple comparison test).

As the regenerating cell wall thickens, it is expected to gain mechanical strength. The mechanical properties of cells at different stages of wall regeneration were evaluated using a single-cell rheometer composed of two parallel plates. A single cell was captured between two custom-made microplates: one rigid and the other flexible, with a calibrated stiffness, functioning like a spring to apply controlled forces to the cell. Uniaxial oscillatory compression was then applied with the flexible plate. During this process, both the deformation of the sample and the applied force were recorded. This approach enabled us to quantify the overall viscoelastic response of the cell, capturing both the storage (K’) and loss (K’’) stiffness as a function of the number of days of wall regeneration and turgor modulation (details in *Materials and Methods*). The storage stiffness K’ relates to the elastic response of the sample, while the loss stiffness relates to the energy dissipation by viscous effects within the sample. An increase in storage stiffness was observed during cell wall regeneration (Fig. 4*C*): K’ increased from 20 ± 11 nN/µm (day 1) on average to 760 ± 195 nN/µm (day 5). Thus, storage stiffness increased by approximately three orders of magnitude over 5 d. The loss stiffness K’’ increased over the 5-d time course, rising by approximately two orders of magnitude from 0.1 nN/µm on day 0 to 10 nN/µm on day 5. Unlike K’, a plateau was observed around day 3 in control condition, suggesting that the loss stiffness stabilizes faster than the elastic stiffness. Although the cultures could be maintained beyond 5 d, eventually leading to full regeneration of the callus tissue, mechanical measurements were limited to the first 5 d. Beyond this period, the onset of cell division introduced multicellularity and structural heterogeneity, thereby interfering with accurate mechanical measurements.

To assess the mechanical properties of the wall in the absence of turgor pressure, viscoelastic measurements were performed in culture medium supplemented with mannitol to impose hyperosmotic stress. The results (Fig. 4) showed that single-cell mechanical assays are highly sensitive to turgor pressure, which is in line with previous studies (36, 44, 45, 48). At each time point, an equal concentration of mannitol was added to the medium to induce plasmolysis, evidenced by detachment of the plasma membrane from the cell wall (arrows, Fig. 4*B*). At all experimental time points (1, 2, 3, 5 d), the measured storage stiffness (K’) (Fig. 4*C*) decreased by approximately one order of magnitude upon plasmolysis. K’ measured under plasmolyzed conditions, reflecting primarily the mechanical properties of the cell wall, increased by about two orders of magnitude over the 5-d regeneration period. This result supports the conclusion that the plant cell wall stiffness increases as the wall thickens.

Our findings demonstrate that turgor pressure is the dominant contributor to the overall storage stiffness of the cell. Quantitative analysis revealed that the cell wall accounts for approximately 10% of the overall elastic stiffness, with the remainder primarily attributable to internal turgor pressure. The loss stiffness (K’’) was minimally affected by hyperosmotic shock (Fig. 4*D*), as plasmolyzed cells exhibited a loss stiffness equivalent to turgid cells. This indicates that the rise of the viscous behavior is primarily due to the regenerating cell wall, which contributed approximately 80% of the measured loss stiffness, with limited input from turgor pressure.

### 1.5. Synthetic Capsules Recapitulate the Mechanical Behavior of Regenerating Cell Walls.

**Fig. 5.**
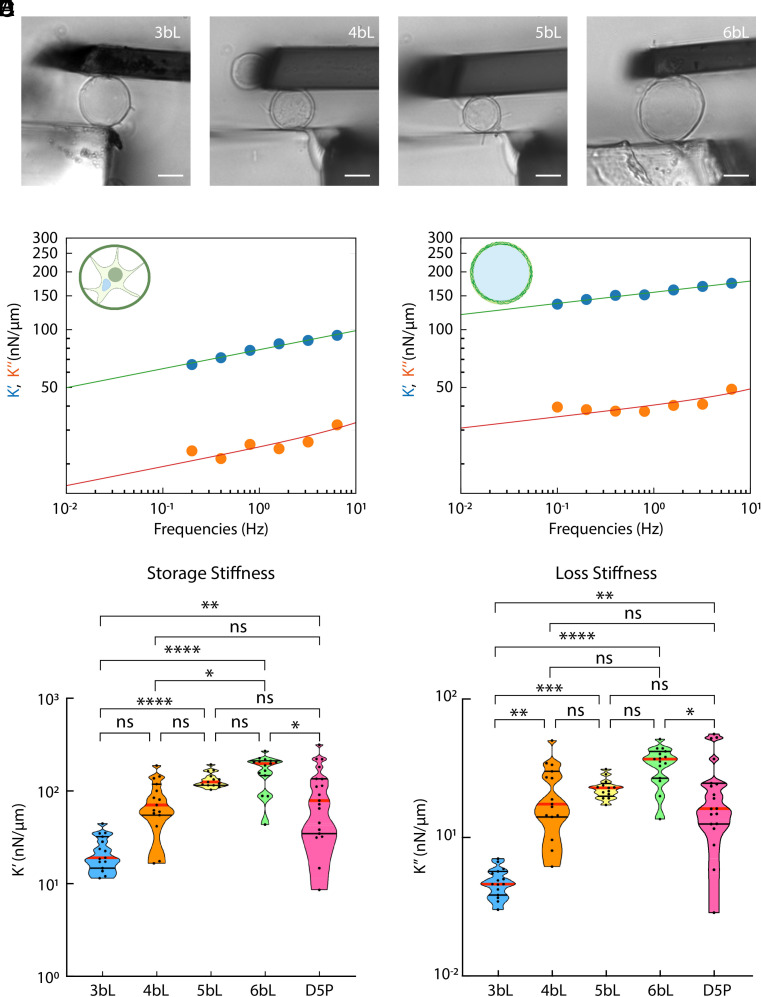
Storage stiffness and loss stiffness of synthetic walls and day 5 plasmolyzed cells. Light microscopy images of the synthetic walls are shown in (*A*) (Scale bar, 10 µm). Storage stiffness (K’, blue) and loss stiffness (K’’, orange) as a function of frequency for day 5 plasmolyzed cell (*B*) and synthetic wall analogues (*C*). Storage stiffness (K’, *D*) and loss stiffness (K’’, *E*) are plotted as a function of thickness for synthetic walls composed of 3 bilayers (3 bL, n = 15), 4 bilayers (4 bL, n = 15), 5 bilayers (5 bL, n = 13), and 6 bilayers (6 bL, n = 15). *P-value < 0.05, **P-value < 0.01, ***P-value < 0.001, ****P-value < 0.0001 Kruskal–Wallis test (Dunn’s multiple comparison test).

To compare mechanical properties, we measured both the synthetic walls and regenerating plant cell walls using the same microplate-based compression device in pure water for the synthetic walls and in BY2 culture medium for the regenerated walls. Oscillatory compression tests revealed that both systems exhibit similar viscoelastic signatures: across the tested frequency range, storage stiffness (K’) consistently exceeded loss stiffness (K’’), indicating that the mechanical behavior is predominantly elastic. In both cases, storage stiffness followed a power-law dependence on frequency, $K^{\prime }=K^{0},f^{\alpha }$, with comparable exponents α = 0.06 ± 0.02 for the synthetic wall and α = 0.12 ± 0.07 for the regenerated wall at day 5 (Fig. 5 *B* and *C*), suggesting similar viscoelastic behavior.

The synthetic capsules also exhibited a monotonic increase in K’ and K’’ as the number of layers increased (Fig. 5 *D* and *E*). Since the thickness of the synthetic wall increases with the number of layers (Fig. 2), the observed rise in elastic stiffness reflects the accumulation of wall material. Notably, the measured values for the synthetic capsules fall within the same range as those of regenerated plant cell walls at day 5 (Fig. 5*D*), highlighting the synthetic wall’s ability to mimic both the elastic and dissipative characteristics of native walls.

### 1.6. Wall Material Quantity Controls Stiffness Evolution in Both Systems.

**Fig. 6.**
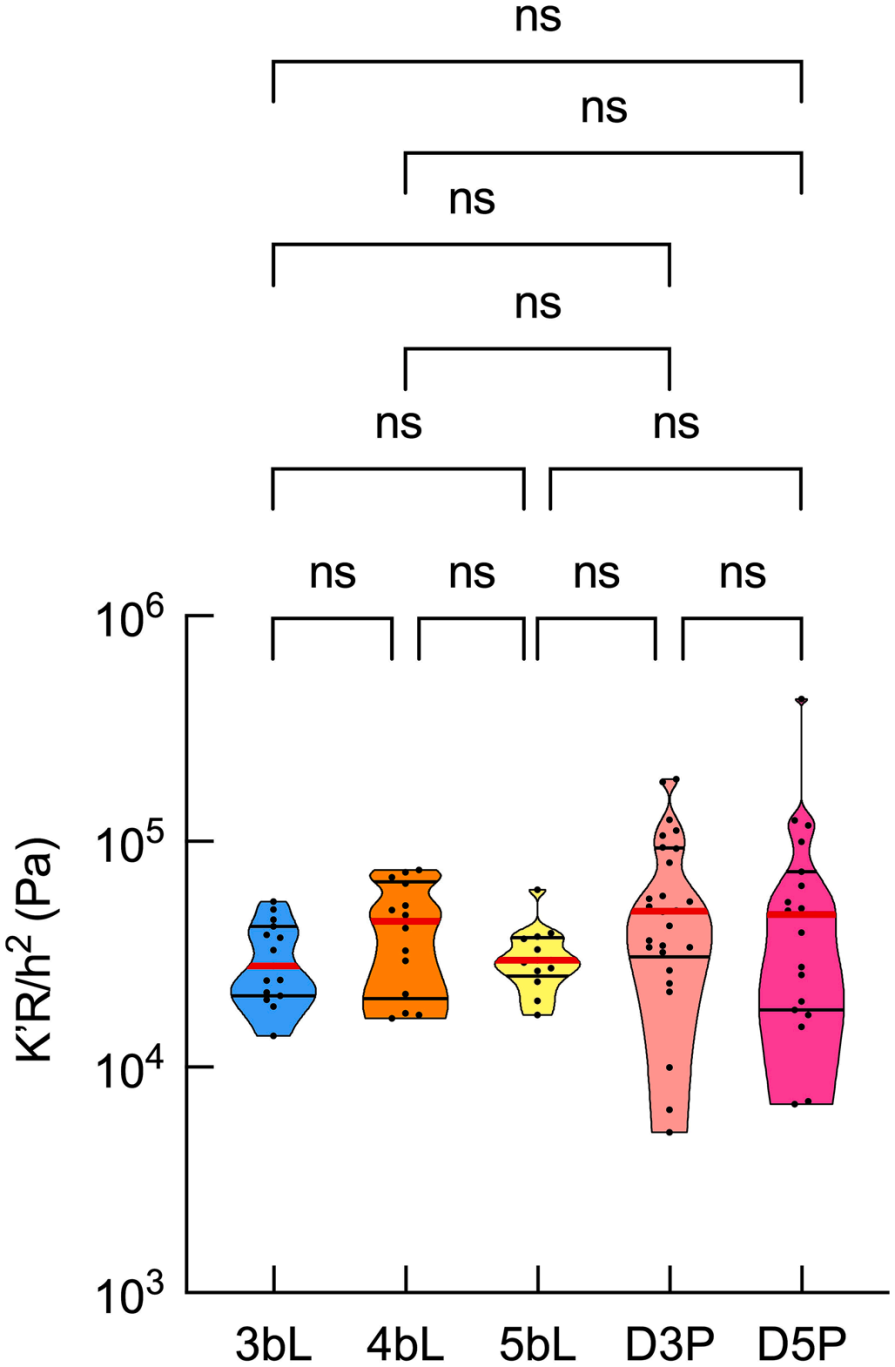
Effective elastic modulus (E’) as a function of thickness. E’ = *K’^.^R/ h^2^* is plotted for synthetic walls composed of 6 bilayers (6 bL, n = 15), 5 bilayers (5 bL, n = 13), 4 bilayers (4 bL, n = 15), and 3 bilayers (3 bL, n = 15), as well as for D3 and D5 plasmolyzed cells (n = 26 and n = 19, respectively). Kruskal–Wallis test (Dunn’s multiple comparison test).

To ensure a fair comparison between the regenerated walls and the synthetic capsules, we estimated the effective Young’s modulus $E^{\prime }$ of the walls from the classical thin-shell theory (49): $E^{\prime }=K^{\prime }R/h^{2}$, where R is the shell radius, and h is the wall thickness measured with TEM (Fig. 2). For both systems, the resulting effective Young’s modulus was independent of the wall thickness (Fig. 6). This reveals that the increase in stiffness reported in the previous sections is due to the material accumulation in the wall through the addition of layers for the synthetic system or through regeneration days for the plant wall. The effective Young’s moduli fall in the same range of values for both systems.

Taken together, these results demonstrate that the synthetic cellulose-pectin capsules not only replicate the magnitude of elastic and loss stiffness of regenerating plant cell walls but also recapitulate the scaling behavior with wall thickness and frequency. While the capsule composition is dominated by pectin and cellulose, the presence of trace contaminants like xylose and mannose and their potential contribution to the measured mechanical properties of the synthetic walls cannot be fully excluded. Nevertheless, under compressive loading at the overall sample scale, synthetic walls recapitulate the key mechanical properties of native primary cell walls and can later serve as an experimental platform for dissecting other features of plant wall mechanics.

## 2. Discussion

Plant cell walls are complex, with highly oriented cellulose microfibrils embedded within a heterogeneous matrix of polysaccharides that are constantly being remodeled. By contrast, the synthetic cell walls offer a simplified and well-controlled platform to test specific hypotheses. These synthetic walls consist of multilayer walls composed mainly of pectin and CNFs. They are structurally static and lack remodeling capacity. Our AFM analysis indicated that CNFs in the synthetic wall are more curved and bundled and less extended compared to the cellulose microfibril cross-lamellate structure observed in regenerated walls on day 3 and 5. Despite these differences in nanoscale architecture, we show that synthetic capsules capture key macroscopic mechanical properties of regenerating cell walls, including storage and loss stiffnesses, under whole-sample compressive testing. However, other methods of mechanical testing, such as nano-scale indentations or creep, may yield different outcomes.

More broadly, similar mechanical properties can be achieved through distinct underlying architectures in other biomimetic materials. For example, natural nacre exhibits remarkable strength and toughness thanks to a highly ordered “brick-and-mortar” architecture of aragonite platelets and softer organic layers (50). Yet, synthetic alumina composites produced by freeze casting can match nacre’s mechanical performance despite a less ordered internal structure (51). Similarly, biomimetic spider silk crosslinked with multiarm polyethylene glycol attains mechanical properties close to natural silk, including tensile strength and toughness despite distinct molecular and fiber arrangement differences (52, 53).

Young’s modulus of plant cell walls ranges from 0.1 to 10 MPa in meristematic tissues (19, 54) and ~0.3 MPa for turgid BY- 2 cells (45). In contrast, the effective Young’s modulus on plasmolyzed BY-2 cells with regenerated walls measured in this study is much lower (~0.06 MPa at day 5). The lower values reported here might indicate that the regenerated wall had not achieved full mechanical maturity at day 5. Stiffness measured on turgid cells (0.76 N/m) is also lower than the stiffness measured previously on intact BY2 cells with Cellular Force Microscopy [4 to 8 N/m, (44)], indicating that turgor pressure is lower in cells with 5 d regenerated walls or that the cell wall is weaker than in intact BY2 cells.

In their construction, the synthetic walls required electrostatic interactions, in particular, cationic modification of CNFs, for Lbl assembly. By comparison, cellulose microfibrils in living plant walls are not positively charged. Nevertheless, negatively charged pectin in native walls associates with cationic partners, for example through homogalacturonan forming pectate coacervates with positively charged extensin proteins (55). This demonstrates that similar mechanical behavior can emerge from different molecular interactions.

In primary cell walls, various types of molecular interactions and bonds between pectin and cellulose (56, 57) and between cellulose and xyloglucan (28) have been reported (Table 1) and certain bonds have been linked to wall strength (28, 58). To which degree such molecular interactions/bonds spontaneously form in our synthetic capsule walls remains uncertain. Nonetheless, the mechanical properties of regenerating walls fall in the same range as those of intact plant cell walls and match those of synthetic capsules. Our findings thus challenge the notion that a specific type of molecular interaction (or bond) between pectin–cellulose and xyloglucan–cellulose is essential for wall strength. This interpretation would be consistent with recent work showing that wall mechanics are primarily sustained by interactions between cellulose fibrils (59).

TEM and POM imaging showed that synthetic walls exhibited a layered and uniform thickness throughout their structure (Fig. 2). However, TEM cross-sections indicated that pectin and CNF layers were not stratified into discrete layers but instead appeared to be partially interpenetrated. In contrast, TEM imaging of native walls revealed variable thicknesses, while POM suggested a layered organization. Lipowczan et al. (60) suggested that plant cell walls are organized in multiple layers of varying thickness, creating elastic strain gradients that reflect both deposition history and remodeling, thereby guiding growth and morphogenesis. While the TEM sample preparation might have generated wall thickness variation in the native wall, the thickness variability identified in our measurements might also reflect wall shaping by local developmental cues and remodeling during wall regeneration. By contrast, synthetic walls exhibit a uniform thickness, reflecting the static and controlled nature of these artificial systems. Nonetheless, at the global cell scale, the architecture of the synthetic walls appears to be sufficient to match the regenerating walls’ mechanical properties assessed with compressive dynamical loading.

Thickness-dependent mechanics are well established for polyelectrolyte multilayer microcapsules. When tested in aqueous environments, microcapsule stiffness scales with wall thickness squared (49). Our quantification shows that the regenerating walls and the synthetic capsules exhibit increasing storage and loss stiffnesses as their walls thicken, showing comparable thickness-dependent mechanical trends. This highlights how the overall amount of material, rather than specific architectural details, governs mechanical behavior in the early days of primary plant cell wall regeneration.

Pectin state (unsubstituted or methyl-esterified) is expected to change during wall regeneration (61). In confocal microscopy, our imaging showed that the PI staining became significant around day 3 after wall regeneration started. Because PI binds to demethylesterified pectin (62), methylesterified pectins might be secreted before day 3, and our results suggest that from day 3 onward they undergo a change in their substitution state, becoming less methylated. AFM imaging highlighted the emergence of a well-organized structure in the cell wall beginning on day 3. This timing coincides with the PI staining becoming significant and with the plateau observed in the loss stiffness, which stabilizes earlier than the storage stiffness (Fig. 4). This temporal correlation suggests that the organized structure with a change in pectin state may be sufficient to confer dissipative mechanical properties to the cell wall. In contrast, the full development of its storage capacity might require further structural reinforcement, potentially mediated by the crosslinking cellulose networks (42).

Confocal microscopy revealed that the distribution of polymers such as cellulose and pectin is more homogeneous in synthetic capsules compared to native cell walls. The spatial heterogeneity in polymer density found in regenerating walls might reflect the dynamic regulation of wall deposition as highlighted in other studies (38, 42). It is also proposed that local polymer organization influences cell morphogenesis and mechanics (63, 64). In addition, nonuniform organization may induce mechanical heterogeneities, as shown by Peaucelle et al. (19), who suggested that local pectin de-methylesterification modulates wall elasticity, triggering organ formation. In contrast, the homogeneous polymer distribution in synthetic capsules reflects the static and controlled nature of these artificial systems. Similar observations have been reported in other capsule types, including alginate–PLL–alginate microcapsules (65) and microcapsules composed of natural polymers (66), both of which display uniformly distributed polymers.

## 3. Conclusion

Plant cell growth depends on the mechanics of the primary wall, which reflect its composition, organization, and remodeling. Comparing regenerating BY2 walls with simplified cellulose–pectin capsules shows that native architectures are not required to reproduce mechanical properties: both systems display thickness-dependent increases in stiffness and viscous dissipation despite distinct nanoscale organizations. This suggests that these cellulose and pectin-based synthetic walls are sufficient to capture mechanical properties of native regenerating primary plant cell walls in compression tests.

Synthetic capsules provide a minimal yet powerful comparative platform that allows independent control of wall composition, thickness, and environmental factors (e.g., hydration, salinity, or temperature), which are difficult to decouple in native systems. This makes it possible to systematically test how specific wall components and interactions affect wall properties under defined conditions.

## 4. Materials and Methods

### 4.1. Materials.

#### 4.1.1. CNF sample preparation.

Never-dried softwood pulp (Nordic Seffle AB, Sweden) were used in reaction with glycidyltrimethylammonium chloride, followed by mechanical disintegration, to generate the cationic (quaternary ammonium groups) CNFs, using a well-documented protocol as described in detail earlier (67). The cationic charge density was (1.2 mmol g^−1^ fiber) for the CNF [conductometric titration, (68)].

### 4.1.2. BY2 Cell Culture and Protoplasting.

BY-2 cells were cultured in BY-2 medium containing 4.67 g/L Murashige and Skoog (MS) basal salt mix without vitamins, 30 g/L sucrose, 0.2 g/L KH_2_PO_4_ (monobasic potassium phosphate), and 0.1 g/L myo-inositol. The pH was adjusted to 6.0 with 1 M KOH prior to autoclaving. After sterilization, 1 mL/L thiamine (0.1 g/100 mL) and 200 µL/L of a 10 mg/mL 2,4-dichlorophenoxyacetic acid (2,4-D) stock solution were added aseptically. For callus culture, the medium was solidified with 7 g/L plant agar. Liquid cultures were initiated by transferring a small piece of 3 wk old callus into 100 mL of liquid BY-2 medium and incubated at 25 °C with shaking at 130 rpm for 1 wk to allow cell proliferation. After 1 wk, 10 mL of the cell suspension were collected and centrifuged at 1,200 rpm for 3 min. The resulting pellet was resuspended in 10 mL of enzymatic solution for protoplast isolation. This solution, prepared in BY-2 medium supplemented with 0.4 M mannitol and thiamine (without hormones), contained 850 mg cellulase R10 (AG Scientific), 850 mg cellulysin (Merck), and 20 mg pectolyase Y23 (Phytotech Labs) per 50 mL. The cell suspension was incubated for 4 h at 60 rpm, room temperature, protected from light with aluminum foil.

Following enzymatic digestion, the suspension was centrifuged at 1,200 rpm for 3 min, and the supernatant was discarded. The pellet was rinsed twice with BY2 medium with 0.4 M mannitol and thiamine, each rinse followed by centrifugation at 1,200 rpm for 3 min and removal of the supernatant. A final wash was performed with BY2 medium supplemented with 0.2 M mannitol and thiamine. The protoplast suspension was then left to rest for 10 min in the dark and filtered through a 45 µm nylon mesh (Fisherbrand) to eliminate debris. After filtration, the suspension was centrifuged again at 1,200 rpm for 3 min, and the supernatant was carefully removed. Protoplasts were finally resuspended in BY-2 medium supplemented with 0.2 M mannitol, thiamine, 200 µL/L of a 10 mg/mL 2,4-dichlorophenoxyacetic acid (2,4-D) stock solution, and 1 mL/L of a 1 mg/mL 6-benzylaminopurine (BAP) stock solution. The protoplast culture was incubated in the dark at 25 °C for 5 d to allow cell wall regeneration.

### 4.1.3. Lbl Assembly of Synthetic Walls.

Apple pectin (0.1 wt%, Sigma, exhibiting 70% esterification) was dissolved in 100 mM NaCl and stirred overnight at room temperature. The CNF (0.05 wt%) was diluted with MilliQ-water and stirred overnight. The pH was adjusted to 7.5 ± 0.2 for both CNF and pectin using 0.1 M NaOH or HCl. The pectin solution was filtered through a 0.8 μm syringe filter (Corning, Germany). The CNF suspension were sonicated for 60 s (Sonics Vibra-Cell, 750 W, 80% amplitude, 1/2″ tip) and then centrifugated for 10 min and the supernatant was collected (sediment discarded). NaCl was then added to the CNF suspension to obtain a 100 mM NaCl content.

Microcapsules were prepared by sequential deposition of apple pectin and cellulose nanofibrils (CNF) onto CaCO_3_ microparticles using a Lbl approach, as described previously (33, 69, 70). CaCO_3_ particles were prepared as described earlier (31, 71). An amount of 25 mg of dry CaCO_3_ particles were dispersed in 4 mL of 100 mM NaCl and sonicated at 40% amplitude for 10 s (Sonics Vibra-Cell, 750 W, 1/2″ tip). The particles were then collected by centrifugation at 5,000 rpm for 1 min. The CaCO_3_ was resuspended in 2 mL of the 0.1 wt% pectin solution (100 mM NaCl, pH 7.5 ± 0.2) and vortexed for 18 min (Vortex Genie, speed 10) to allow pectin to adsorb on the CaCO_3_ particles. After centrifugation, the supernatant was removed, and the particles were washed three times by resuspending in 2 mL of 100 mM NaCl solution, vortexing for 1 min, and centrifuging under the same conditions. Next, the CNF layer was deposited by incubating the washed particles in 2 mL of the 0.05 wt% CNF suspension (100 mM NaCl, pH 7.5 ± 0.2) and vortexed for 18 min, followed by the same washing procedure. This deposition cycle was repeated to obtain the desired number of bilayers (3 to 6). Following Lbl assembly, the coated CaCO_3_ particles were rinsed with 100 mM NaCl for 1 time, then with MilliQ-water for 5 times and then dried at 50 °C overnight. Prior mechanical testing, dried particles were rehydrated in MilliQ water for 10 to 60 min and then incubated in 50 mM citric acid at least 1 h to allow the CaCO_3_ core to dissolve. Zeta potential experiments on pectin or cationic CNFs in a solution with pH 2.3 for 1 h (adjusted with HCl), then adjusted to neutral pH (NaOH) and dialyzed against MilliQ water (2 d) confirmed that acidic treatment does not affect the surface charge. The capsules were then thoroughly washed with MilliQ water and stored at room temperature in water prior to further characterization. The final pH of the water solution in which the capsules are tested is around 6.0.

### 4.2. Characterization.

#### 4.2.1. Atomic force microscopy.

AFM imaging was performed as previously described in ref. 72. Protoplasts were washed in BY-2 medium, pelleted by centrifugation, and a small volume of cells was deposited on silane-coated glass microscope slides and allowed to dry completely. At the time of scanning, the protoplasts were rehydrated in ddH2O and washed until no further debris was removed. The Lbl capsules were similarly rehydrated on glass slides at the time of AFM scanning. Capsule cores were removed as described prior and after washing, hydration was maintained with ddH2O throughout the scanning process. AFM topography images were captured on a Dimension Icon AFM (Bruker, CA) using a calibrated Scanasyst-Fluid + probe (spring constant of 0.7 N/m, nominal tip radius of 2 nm; Bruker). 2 µm × 2 µm images were captured in QNM PeakForce Tapping mode (in fluid) at a resolution of 1,024 pixels/line. All images in figures are based on the Height Sensor AFM channel, first-order plane fit, and fifth order flattened using Nanoscope Analysis V 2.0 (Bruker) software, to show details.

#### 4.2.2. Transmission electron microscopy.

BY-2 cells were fixed daily in a 1% (v/v) glutaraldehyde (GA) solution prepared in BY-2 medium supplemented with 0.2 M mannitol. The fixation was carried out overnight at 15 °C with gentle agitation. The following day, cells were centrifuged for 2 min at 1,300 rpm, the supernatant was removed, and the pellet was resuspended in 500 µL of BY-2 medium containing 0.2 M mannitol.

The cell suspension was then transferred into sealed capsules, which were placed in Eppendorf tubes and centrifuged again for 2 min at 1,300 rpm. After removal of the residual supernatant, cells were overlaid with 500 µL of 20% gelatin (Oetker) prepared in 0.2 M mannitol and maintained at ~37 to 40 °C to remain liquid. Capsules were left at room temperature until the gelatin solidified completely. Additional fixation was done with 2.5% GA. For the synthetic walls, core-removed capsules in water were fixed with 2.5 wt% GA for 2 h. After fixation, all samples were transferred to EtOH absolute in steps (50, 70, 90, and ≥99.5 vol%) and embedded in epoxy (Epoxy embedding medium kit Sigma Aldrich) in polyethylene molds (BEEM capsules). The resin was hardened at 45 °C (12 h), followed by 60 °C (24 h). Samples were cut with a Leica Ultramicrotome (EM FC7). The ultrathin sections were stained with uranyl acetate (5% in water) for 45 min. TEM micrographs were attained with a Thermo Scientific Talos L120C transmission electron microscope (acceleration voltage of 120 kV). The thickness of the wall was measured with ImageJ.

#### 4.2.3. POM.

Was attained using crossed linear polarizers in transmission mode and an Axio Vert.A1 Light Microscope (Carl Zeiss, Germany) equipped with a Zeiss AxioCam 305 color camera [Zeiss Zen 2.6 (blue edition) software], and a 63×/0.65 LD A-plan objective.

#### 4.2.4. CLSM.

For each day of cell wall regeneration, protoplasts were stained with a final concentration of 0.02% (w/v) Calcofluor White Brightener 28 (CFW) from a 0.2% stock solution and 0.01% propidium iodide (PI) prepared from a 1 mg/mL stock solution. Stained cells (50 µL) were mounted between a Superfrost microscope slide and a 1.5 thickness coverslip, separated by a spacer, and immersed in immersion oil (refractive index 1.334), compatible with the oil immersion objective used for imaging. Synthetic cell walls, with CaCO_3_ cores dissolved, were stained with 0.001% CFW (w/v) or 0.003% PI (w/v) and were similarly mounted for imaging.

Imaging of regenerated and synthetic walls was performed on Zeiss LSM 780 confocal microscope(s) equipped with a 63×/1.2 NA Olympus oil immersion objective, or a 63×/1.4 oil DIC M27 immersion objective (for the PI-stained synthetic cell walls). For dual-stained plant cells, single channels were acquired sequentially using “channel per track” mode to avoid cross-talk. The CFW signal was excited with the 405 nm laser at 2% power. PI was excited with the 543 nm laser at 27.9% power. Acquisition parameters were as follows: zoom 1.5, image size 512 × 512 pixels, pixel size 0.18 µm, and Z-step of 0.38 to 0.5 µm. CFW-stained synthetic walls were imaged with a 405 nm laser at 3% power (3bL, 4bL) or 2% power (5 bL), using the following parameters: zoom 1, image size 1,024 × 1,024 pixels, pixel size 0.13 µm × 0.13 µm and Z-step of 0.5 µm. PI-stained synthetic cell walls were imaged with a 514 nm laser at 25% power with the same acquisition parameters.

#### 4.2.5. Gas chromatography–mass spectrometry.

The monosaccharide composition of the cell wall was analyzed. Three biological replicates, each consisting of approximately 10^5^ to 10^6^ BY2 cells, were subjected to three successive washes in an ascending ethanol gradient (50%, 80%, and 100%). A final wash was performed with acetone, after which the samples were dried overnight at room temperature. To remove residual starch, the dried material was incubated overnight at 37 °C with α-amylase from **Aspergillus* oryzae* (Sigma, ref. A9857) in 200 µL of 100 mM ammonium acetate buffer (pH 5.15). After digestion, the supernatant was discarded and the pellet was washed twice with 500 µL of ammonium acetate buffer and one time ethanol 70% before drying.

For hydrolysis, the dried regenerated cell walls and synthetic walls were resuspended in 400 µL of freshly prepared trifluoroacetic acid (TFA, 2,5 M) and incubated at 120 °C for 1 h in 1.5 mL screw-cap tubes. This treatment specifically hydrolyzes the TFA-soluble polysaccharides (mainly hemicelluloses and pectins) into their constituent monosaccharides, while crystalline cellulose remains intact. After centrifugation (10 min), 10 μL of the supernatant was transferred and dried at room temperature using a speed-vacuum concentrator.

For derivation, 10 μL of 20 mg mL^−1^ methoxyamine in pyridine were added to the samples and the reaction was performed for 90 min at 28 °C under continuous shaking in an Eppendorf thermo- mixer. 50 μL of N-methyl-N-trimethylsilyl-trifluoroacetamide (MSTFA) (Aldrich 394866^–10^ × 1 mL) were then added and the reaction continued for 30 min at 38 °C. After cooling, 45 μL were transferred to an Agilent vial for injection. Four hours after the end of derivatization, the whole sample series was injected in splitless mode. Five different standard mixes were injected at the beginning and samples were randomized. An alkane mix (C10, C12, C15, C19, C22, C28, C32, C36) was injected for external retention index (RI) calibration. Injection volume was 1 μL. The instrument was an Agilent 7890A gas chromatograph coupled to an Agilent 5977B mass spectrometer.

Gas chromatography–mass spectrometry (GC-MS) conditions as well as data processing were performed as described in ref. 73. In this study, peak areas were normalized to myo-inositol (internal standard) and to the dry weight of the samples.

#### 4.2.6. Mechanical testing.

Mechanical characterization of synthetic capsules and BY2 cells was performed with a parallel plate device as described previously in refs. 36 and 37. More precisely, we measured the storage stiffness (K’: elastic-like properties) and loss stiffness (K’’: dissipative, viscous-like features) at the whole cell scale by performing dynamical oscillations tests in the range of 0.1 to 6.4 Hz. The cell mechanical behavior as a function of the frequency can thus be represented by K’(f) and K’’(f).

In practice, a single cell or capsule was captured between two custom-made glass microplates under a bright-field microscope. One microplate is stiff compared to the sample while the other one is flexible with a calibrated stiffness k (1-147 nN/μm). A sinusoidal displacement *D*(*t*) = *D*_0_*^iωt^* was imposed to the basis of the flexible microplate, leading thus to a sinusoidal compressive force applied on the cell. To determine the cell deformation, the displacement *d*(*t*) = *d*_0_*e^iωt^*^+^*^ϕ^* of the flexible microplate, (with *ϕ* the phase shift between D and d) was measured through a S3979 position-sensitive detector (resolution ∼200 nm). The instantaneous force can be calculated *F*(*t*) = (*D*(*t*)–*d*(*t*)). Both *D*(*t*) and *d*(*t*) were well fitted by a sinus function. The elastic stiffness K’ and loss stiffness K’’ were then given by

[1] $$K^{\prime }=K\left[\frac{D\left(\omega \right)}{d\left(\omega \right)}\left(\cos\left(\phi \left(\omega \right)\right)-1\right)\right],$$

[2] $$K^{\prime \prime }=-K\left[\frac{D\left(\omega \right)}{d\left(\omega \right)}\sin\left(\phi \left(\omega \right)\right)\right].$$

All results presented were performed for strains between 1% and 10% to avoid a nonlinear response due to too large strains. For all tested cells and synthetic walls, both K’ and K’’ exhibited a frequency dependence following a weak power-law behavior, described by

[3] $$K^{\prime }=\left(f\right)=K_{0}^{\prime }f^{\alpha },$$

[4] $$K^{\prime \prime }\left(f\right)=K_{0}^{\prime \prime }f^{\alpha }+2\pi \eta f.$$

In the results section of the paper, the characteristic storage stiffness K’ and loss K’’ stiffness at the reference frequency f_0_ = 1 Hz are reported (corresponding respectively to K’_0_ and K’’_0_ extracted from the power law fit after frequency sweep on each cell).

Cells between the microplates were visualized under bright light illumination with a Plan Fluotar L 63×/0.70 objective and a Lumenera digital CCD camera (Infinity3, Lumenera, Canada). Vibration isolation was achieved by a TS-150 active antivibration table (HWL Scientific Instruments, Tübingen, Germany).

The storage (K’) and loss (K”) stiffnesses were measured on individual cells or individual bL capsules using the microplate system. For each experimental condition and for each day of cell wall regeneration, measurements were performed on a total of 20 to 30 cells, distributed over two independent experiments with 10 to 15 cells per experiment to ensure reproducibility.

#### 4.2.7. Mannitol treatment for plasmolysis.

Initially, turgid cells were placed in 10 mL of BY-2 medium supplemented with 0.2 M mannitol, corresponding to an osmolarity of approximately 425 mOsmol, measured with an osmometer. This condition represents the turgid state of the cells. To induce plasmolysis, 4 mL of BY-2 medium containing 0.7 M mannitol (osmolarity approximately 1,020 mOsmol) was added, resulting in a final medium osmolarity of about 630 mOsmol. This hyperosmotic shock triggers cell plasmolysis. Mechanical measurements were conducted on plasmolyzed cells 20 min after the hyperosmotic treatment to allow stabilization of their physiological state.

### 4.3. Statistical Analysis.

Statistical analyses were performed using GraphPad Prism 10. Data normality was assessed using the Shapiro–Wilkand D’Agostino–Pearson tests. Since the data did not follow a normal distribution, nonparametric tests were applied: the Kruskal–Wallis/Dunn’s test for multiple comparisons or the Mann–Whitney test for single comparisons.

## Supplementary Material

Appendix 01 (PDF)

## Data Availability

Raw data are available on Zenodo (74).
