# Supplementary material for: Synthetic pectin–cellulose nanofiber capsule recapitulates the mechanical properties of a regenerating plant cell wall
Source: Proc Natl Acad Sci U S A. 2026 Jun 1;123(23):e2528515123. doi: 10.1073/pnas.2528515123 (PMC13250591; doi:10.1073/pnas.2528515123)
Supplement: Supplementary file 1 — Appendix 01 (PDF) [file pnas.2528515123.sapp.pdf]

SUPPLEMENTAL INFORMATION

Synthetic pectin-cellulose nanofiber capsule recapitulates the mechanical properties of a regenerating plant cell wall.

Cyril Grandjean[1], Ravi Shanker[2], Sarah A. Pfaff[3], Anran Mao[2], Jordi Chan[4], Sophie Asnacios[1], Atef Asnacios[1], Sulin Zhang[3], Daniel J. Cosgrove[3], Enrico Coen[4], Anna J. Svagan*[2], Pauline Durand-Smet*[1]

[1] Université Paris Cité, CNRS UMR 7057, Laboratoire Matière et Systèmes Complexes, Paris, France

[2]Royal Institute of Technology, KTH, Teknikringen 56-58, SE-100 44, Stockholm, Sweden

[3]Pennsylvania State University, Department of Biology, University Park, Pennsylvania 16802, United States

[4]John Innes Centre, Department of Cell and Developmental Biology, Norwich Research Park, Colney Lane, Norwich NR4 7UH, UK

*Corresponding authors

Table S1. Quantification of monosaccharides for regenerated and synthetic walls Values are presented as mean ± standard deviation. Only non-cristalline components are analysed.

Monosaccharide	Synthetic wall (ng/mg DW)	Synthetic wall (mol%)	Regenerated wall Day5 (mol%)
Arabinose	33.49 ± 1.05	1.35 ± 0.59	20.17± 1.57
Fucose	–	–	–
Galactose	382.78 ± 92.45	13.62 ± 8.90	22.22 ±4.19
Galacturonic acid	1899.07 ± 1063.90	50.02 ± 16.40	3.66 ± 0.67
Glucuronic acid	8.01 ± 6.42	0.19 ± 0.13	0.00 ± 0.00
Glucose	847.41 ± 286.04	26.24 ± 4.57	27.58 ± 6.24
Mannose	83.76 ± 18.41	2.70 ± 0.81	21.38 ± 2.13
Rhamnose	55.68 ± 18.66	1.92 ± 0.46	1.50 ± 0.33
Xylose	94.77 ± 22.47	3.94 ± 2.23	3.47± 1.04

*n.a. = not attained

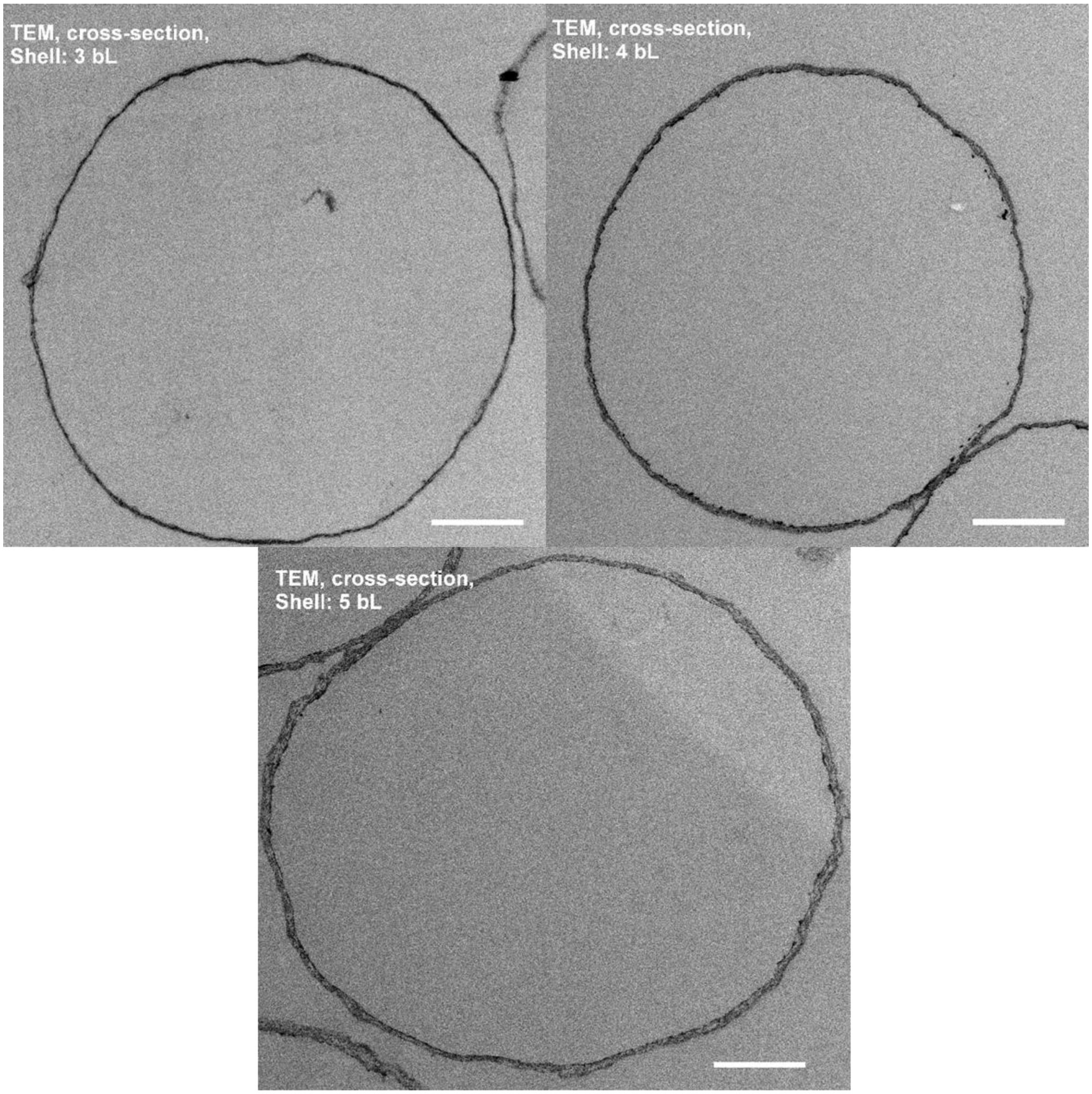

Supplementary Fig 1. TEM cross-sections of synthetic analogues with walls made from 3 bL, 4 bL and 5 bL. Scale bars=2 µm.

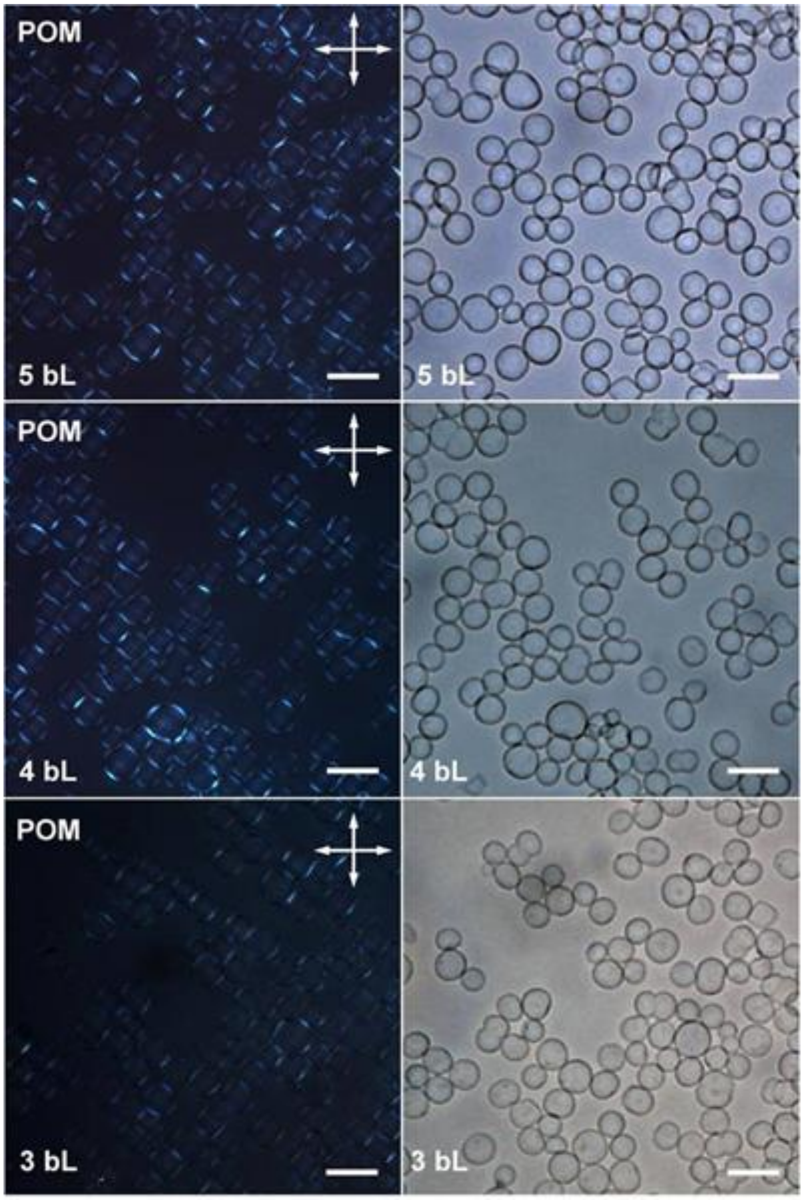

Supplementary Fig 2. Polarized optical microscopy (POM) and Bright field images of synthetic walls made from 3 bL, 4 bL and 5 bL of pectin and CNF. Scale bars=20 μm.

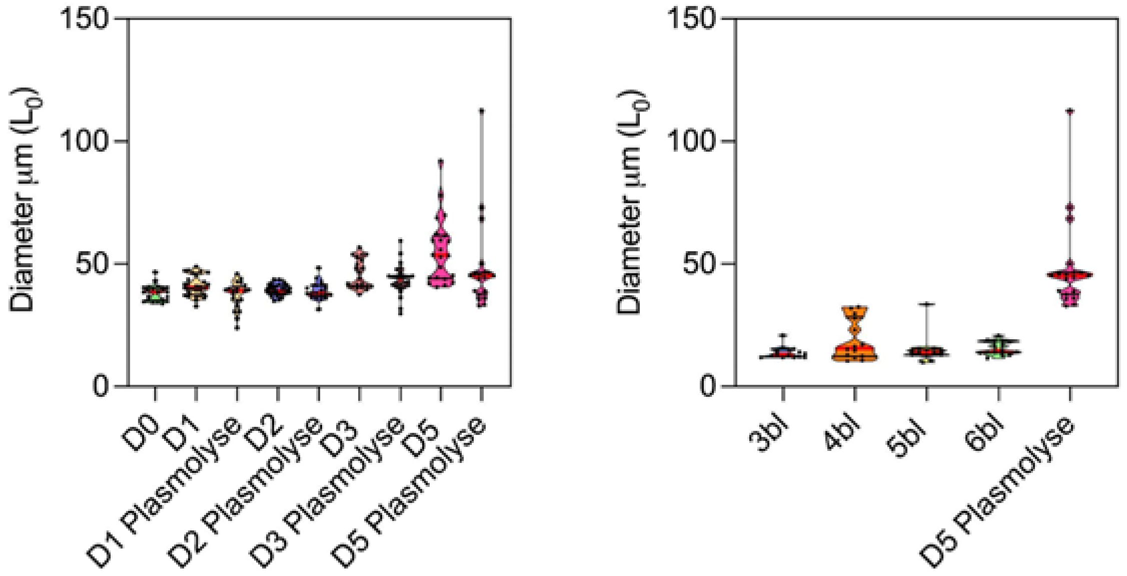

Supplementary Fig 3. The diameter of the cells and synthetic analogues, measured during microplate-based mechanical assays, was used to define the initial contact length (L_0) for the calculation of mechanical parameters. Notably, plant cells exhibit diameters at least three to four times greater than those of the synthetic analogues.

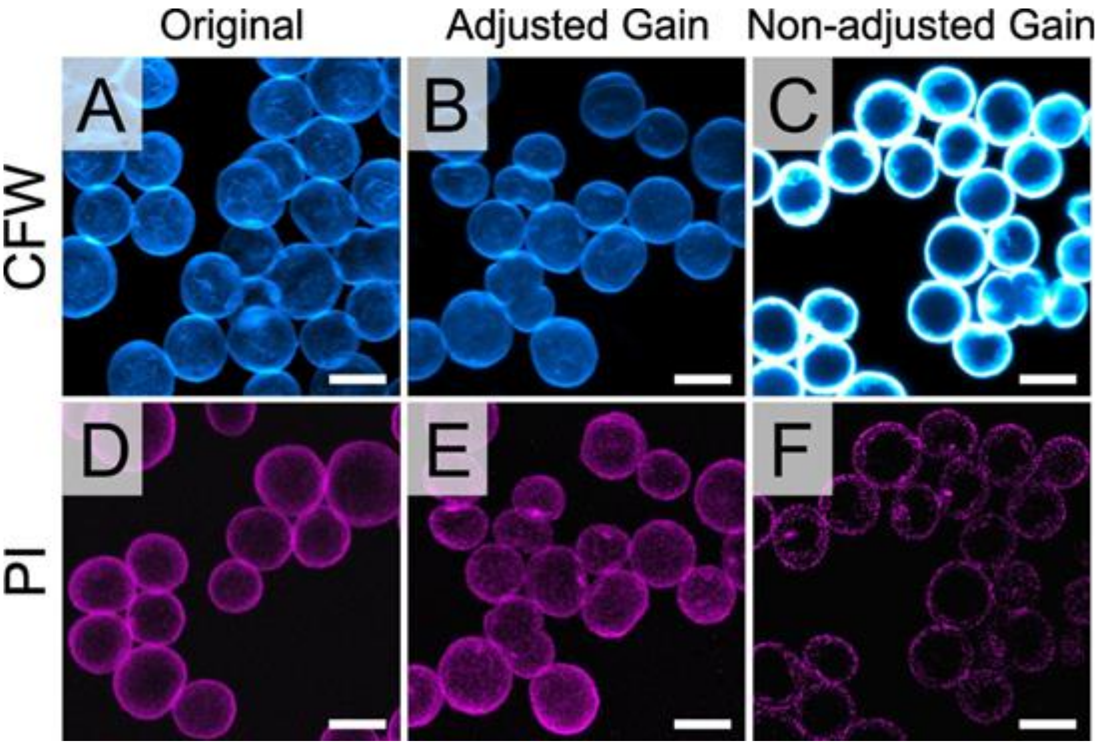

Supplementary Fig 4. CLSM images of synthetic analogues with 5bL. Panels A and D are reproduced from the main manuscript and were acquired using a Zeiss 780 confocal microscope in Sweden. Panels B, C, E, and F were acquired using the same microscope as the one used for the regenerated walls in Fig. 3 of the main manuscript. For panels C–F, images were captured using the same gain settings as those used for the regenerating cells, whereas for panels B and E the gain was adjusted. The cellulose was stainend with Calcofluor White (0.001 % in panel A and 0.02 % in panel B and C) and pectin was stained with Propidium Iodine (0.003 % in panel D and 0.01 % in panel E and F). Scale bars = 10 µm.

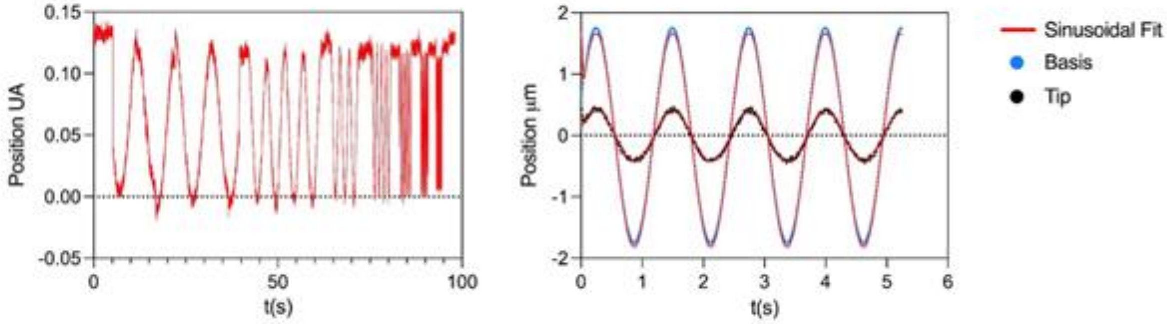

Supplementary Fig. 5: Representative raw signals recorded for a plant cell with regenerated wall. Left, Flexible microplate tip position. The raw signal (red) displays the displacements imposed for different oscillatory frequencies (0.1, 0.2,0.4,0.8,1.6, 3.2 and 6.4 Hz). Right, Sinusoidal oscillations of the microplate at a frequency of 0.8 Hz. Blue dots represent the displacement imposed at the basis of the flexible microplate and black dots correspond to the displacement measured at the tip of the flexible microplate.

The red curves show the sinusoidal fit performed. All positions are expressed in micrometers (µm).

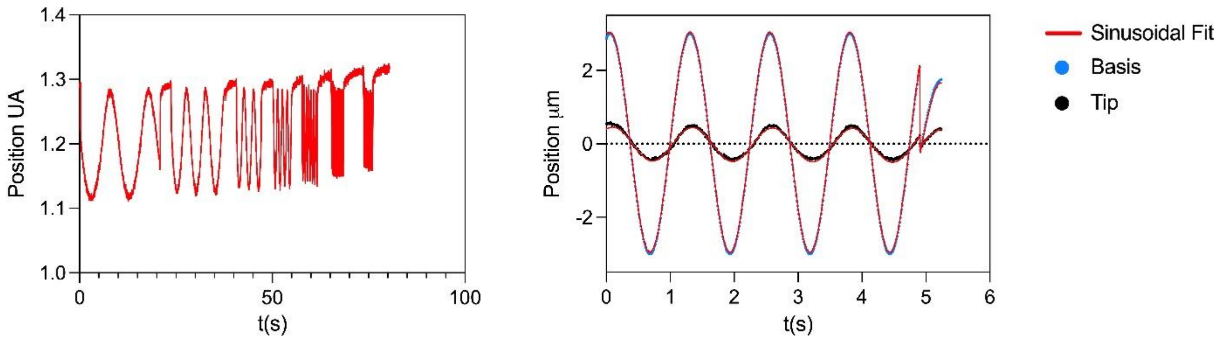

Supplementary Fig. 6: Representative raw signal recorded for a synthetic wall. Left, Flexible microplate tip position. The raw signal (red) displays the displacements imposed for different oscillatory frequencies (0.1, 0.2,0.4,0.8,1.6, 3.2 and 6.4 Hz). Right, Sinusoidal oscillations of the microplate at a frequency of 0.8 Hz. Blue dots represent the displacement imposed at the basis of the flexible microplate and black dots correspond to the displacement measured at the tip of the flexible microplate. The red curves show the sinusoidal fit performed. All positions are expressed in micrometers (µm).
